# Maternal Physiological Variations Induced by Chronic Gestational Hypoxia: ^1^H NMR-Based Metabolomics Study

**DOI:** 10.3390/molecules27228013

**Published:** 2022-11-18

**Authors:** Jing-Xian Xie, Qiu-Fang Chen, Yan-Feng Fan, Yao Qin, Xue-Qin Zhang, Hong-Xiu Zhong

**Affiliations:** 1 Department of Obstetrics, Women and Children’s Hospital, School of Medicine, Xiamen University, Xiamen 361000, China; 2 Science and Education Division, Women and Children’s Hospital, School of Medicine, Xiamen University, Xiamen 361000, China; 3 Department of Nutrition Clinic, Women and Children’s Hospital, School of Medicine, Xiamen University, Xiamen 361000, China

**Keywords:** pregnancy, chronic hypoxia, physiological variations, nuclear magnetic resonance, metabolomics

## Abstract

Metabolomics have been widely used in pregnancy-related diseases. However, physiological variations induced by chronic hypoxia during pregnancy are not well characterized. We aimed to investigate physiological variations induced by chronic hypoxia during pregnancy. A Sprague–Dawley (SD) pregnant rat model of chronic hypoxia was established. Plasma and urine metabolite profiles at different stages of the pregnancy were detected by ^1^H NMR (nuclear magnetic resonance). Multivariate statistical analysis was used to analyze changes in plasma and urine metabolic trajectories at different time-points. We identified hypoxia-induced changes in the levels of 30 metabolites in plasma and 29 metabolites in urine during different stages of pregnancy; the prominently affected metabolites included acetic acid, acetone, choline, citric acid, glutamine, isoleucine, lysine, and serine. Most significant hypoxia-induced changes in plasma and urine sample metabolites were observed on the 11th day of gestation. In summary, chronic hypoxia has a significant effect on pregnant rats, and may cause metabolic disorders involving glucose, lipids, amino acids, and tricarboxylic acid cycle. Metabolomics study of the effect of hypoxia during pregnancy may provide insights into the pathogenesis of obstetric disorders.

## 1. Introduction

Oxygen microenvironmental homeostasis during pregnancy is essential for fetal growth and development [1]. Intrauterine hypoxia affects the maternal physiological state [2,3,4] and may lead to impaired blood supply to the placenta [5]. Moreover, intrauterine hypoxia is also hazardous for the fetus [6], and may lead to intrauterine growth retardation [7,8]. Abnormal placental circulation induced by hypoxia has been implicated in the pathogenesis of many serious obstetric complications. For example, placental hypoxia can induce uterine artery constriction, leading to increased uterine vascular pressure and increased risk of pre-eclampsia [9]. Fetal distress is a commonly encountered obstetric complication in clinical settings and is one of the main causes of adverse perinatal outcomes and perinatal death [10]. Insufficient maternal oxygen supply during pregnancy is the most important cause of fetal distress. Therefore, early diagnosis of intrauterine hypoxia is a key imperative to avoid adverse neonatal outcomes. However, currently, there is no gold standard for the diagnosis of intrauterine hypoxia [11]. 

Metabolomics is a systems biology-based approach that is used to study complex organismal biochemical processes in response to various biological factors at the metabolic scale. Metabolomics analysis of biological fluids or tissues has helped identify physiologic or pathologic markers of the underlying metabolic changes during pregnancy. Indeed, use of maternal blood, urine, amniotic fluid, and umbilical cord blood to characterize relevant prenatal diseases (such as preeclampsia, fetal malformations, poor pregnancy outcome, and preterm delivery) is a contemporary research hotspot [12,13,14].

The objective of this study was to analyze the metabolite changes in plasma and urine in a rat model of hypoxia during pregnancy. By characterizing the hypoxia-induced maternal physiological changes during the early, middle, and late stages of gestation, we sought to identify the potential molecular mechanisms of the effects of gestational hypoxia on the maternal physiology. Our work provides an overview of the maternal metabolic trajectories during gestational hypoxia and the underlying metabolic mechanisms. Furthermore, it provides theoretical data for further study of the effects of gestational hypoxia and may provide insights for early intervention against gestational hypoxia.

## 2. Results

### 2.1. HNMR-Based Metabolomics Analysis of Plasma

#### 2.1.1. Plasma Metabolic Profiling during Different Periods of Pregnancy under Hypoxic Conditions

**Figure 1:**
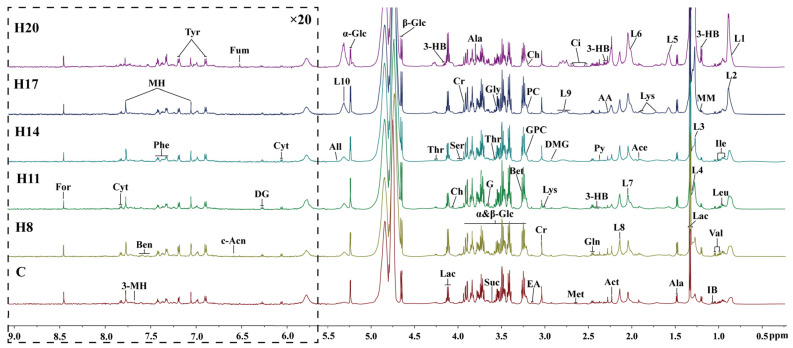
600 MHz ^1^H NMR spectra (δ0.5–9.0) of serum’s samples obtained from C group, and H group at different periods (D8, D11, D14, D17, and D20). The spectra in the region δ5.5–9.5 were magnified 20 times compared with the region δ0.5–5.5 for the purpose of clarity. Ala, alanine; 3-HB, 3-Hydroxybutyrate; 3-MH, 3-Methylhistidine; AA, acetoacetate; Ace, acetate; Act, acetone; All, allantoin; Ben, benzoate; Bet, betaine; c-Acn, cis-aconitate; Ch, choline; Ci, citrate; Cr, creatine; Cyt, cytidine; DG, deoxyguanosine; DMG, N,N-Dimethylglycine; EA, ethanolamine; For, formate; Fum, fumarate; G, glycerol; Gln, glutamine; Gly, glycine; GPC, glycerophosphocholine; IB, isobutyrate; Ile, isoleucine; Leu, leucine; L1, L3, LDL; L2, L4, L5, VLDL; L6, L7, L8, L9, L10, lipid; Lac, lactate; Lys, lysine; Met, methionine; MH, 1-Methylhistidine; MM, methylmalonate; NAG, N-Actyl-glycoprotein; PC, phosphocholine; Phe, phenylalanine; Py, pyruvate; Sar, sarcosine; Ser, serine; Thr, threonine; Tyr, tyrosine; Val, valine; *α*-Glc, *α*-Glucose; β-Glc, β-Glucose.

Concentrations of 46 plasma metabolites were measured using ^1^HNMR spectrogram Library (HMDB). As shown in Figure 1, the plasma profiles in the hypoxia and control group were similar at different stages; however, there were some differences in the concentrations of metabolites within the hypoxia group at different stages, such as relatively high levels of lactic acid and acetic acid (related to glucose metabolism), the intermediate products of TCA cycle (citric acid and succinic acid), and various amino acids such as glycine, alanine, and serine. This indicated that the duration of exposure to hypoxia may have different effects on metabolism. However, owing to the limited accuracy of ^1^HNMR, multivariate statistical analysis was applied to analyze the differences in metabolic data at different time-points in the hypoxia group.

#### 2.1.2. Plasma Metabolic Trajectories at Different Stages of Pregnancy under Hypoxic Conditions

**Figure 2:**
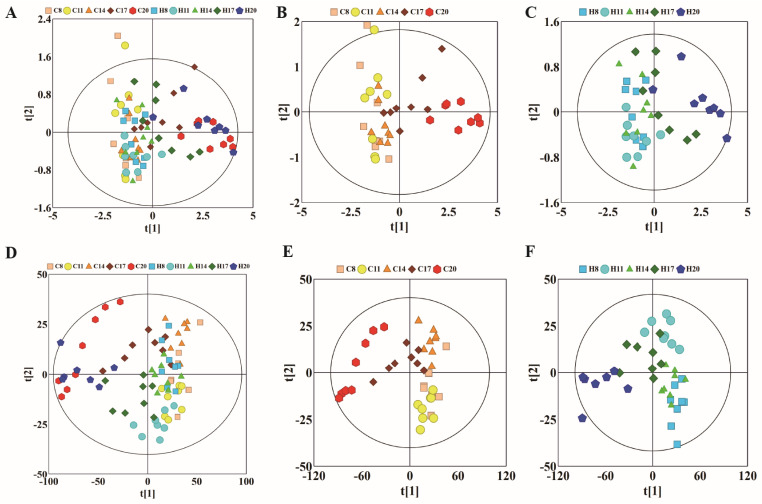
PCA and corresponding PLS-DA score plots derived from ^1^H NMR spectra of plasma samples obtained from different groups at different gestation period. (**A**) PCA score plots of control group and hypoxia group; (**B**) PCA score plots of control group; (**C**) PCA score plots of hypoxia group. (**D**–**F**) corresponding PLS-DA score plots of (**A**–**C**). (C: control group; H: hypoxia groups).

To observe the changes in metabolic trajectories and explore the possible outliers, we performed principal component analysis (PCA) of ^1^HNMR data of plasma of pregnant rats at different stages of pregnancy (Figure 2). In Figure 2A, the first and second principal components (PCs) accounted for 91.7% of the total variance between hypoxia group and control group. Among them, the data of D8, D11, and D14 showed varying degrees of overlap between the control and hypoxia groups, while data of D17 showed a tendency to separate. More obvious degree of separation was found in data of D20. Subsequently, we used PCA to analyze the control group (warm color system, Figure 2B) and hypoxia group (cold color system, Figure 2C), respectively. The first and second principal components accounted for 92.3% and 92.6% of the total variance, respectively. The control group and hypoxia group showed the same change rule on the PC1 axis, which was similar to the overall PCA. There was some overlap on D8, D11, and D14 of pregnancy, while the data was distinctly separated on D17 and D20. Moreover, there was a good distinction between D17 and D20. These results suggested similar metabolic changes in the hypoxia group and control group at different stages of pregnancy. PLS-DA revealed clear separation between groups while partial overlap was observed between the hypoxia and control groups on any particular time-point of pregnancy (Figure 2D–F).

#### 2.1.3. Plasma Physiological and Metabolic Changes Induced by Hypoxia during Pregnancy

**Figure 3:**
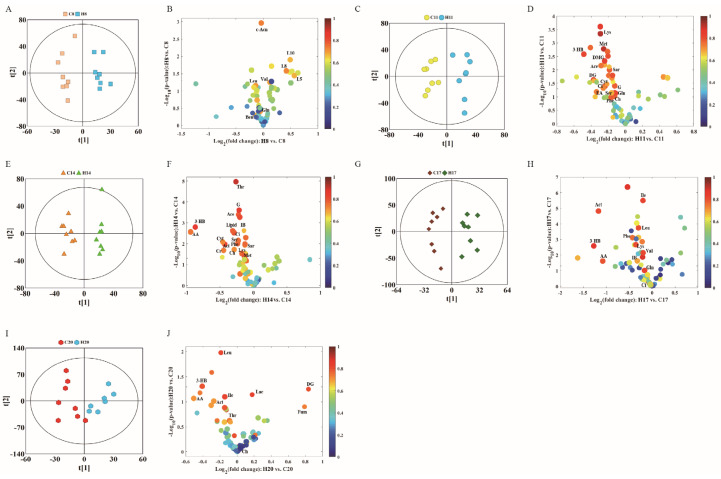
OPLS-DA score plots (**left** panel) derived from ^1^H NMR spectra of plasma samples and corresponding volcano plots (**right** panels) obtained from different groups and cross validation (**lower** panel) by permutation test (*n* = 200). (**A**,**B**) D8; (**C**,**D**) D11; (**E**,**F**) D14; (**G**,**H**) D17; and (**I**,**J**) D20. The color map shows the significance of metabolites variations between the two classes. Peaks in the positive direction indicate metabolites that are more abundant in the groups in the positive direction of first principal component. Consequently, metabolites that are more abundant in the groups in the negative direction of first primary component are presented as peaks in the negative direction. Keys of the assignments were shown in Figure 1.

To further study the effects of hypoxia on the physiology and metabolism of pregnant rats, we subsequently performed OPLS-DA to analyze the NMR data of the two groups. We conducted OPLS-DA of data of D8, D11, D14, D17, and D20 in the control and hypoxia groups (Figure 3A,C,E,G,I), combined with volcano plots (Figure 3B,D,F,H,J) to determine significantly different metabolites.

In the volcanic maps, significantly different metabolites were screened by three restrictive conditions: *p* < 0.05, |r| > 0.5, and VIP higher than the first 10%. Therefore, the point of significantly different metabolites was above the volcanic map area (i.e., above the horizontal threshold dotted line *p* = 0.05). In addition, the point was larger in size and warmer in color. The differences in the metabolite profile between hypoxia group and control group are summarized in Table 1.

Compared with the control group, hypoxia group showed changes in 30 different metabolites in the plasma during different stages of the pregnancy. The metabolite changes can be summarized as follows: (1) The level of citric acid (an intermediate product of TCA) decreased, while that of fumaric acid increased on D20. (2) The levels of acetoacetic acid, 3-hydroxybutyric acid, and acetone showed a downward trend, indicating increased metabolism of ketones. (3) The level of branched-chain amino acids leucine, and valine showed a complex pattern of change: increased on D8, leucine decreased on D17 and D20, valine decreased on D17, while isoleucine only decreased on D17 and D20. The levels of other amino acids such as lysine and methionine showed a downward trend during different stages of gestation. (4) Lactic acid level increased only on D20, and there was no significant difference at other time-points. Acetic acid level significantly decreased on D11 and D14. (5) In addition, in choline metabolism, creatine levels on D14 and ethanolamine levels on D11 showed a significant decrease. Serine and creatine levels significantly decreased on D11 and D14. (6) Low density lipoprotein (LDL) and very low density lipoprotein (VLDL) significantly decreased on D20, while VLDL significantly increased on D8.

### 2.2. ^1^HNMR-Based Metabolomics Analysis of Urine

#### 2.2.1. Urine Metabolic Profiling during Different Stages of Pregnancy under Hypoxic Conditions

**Figure 4:**
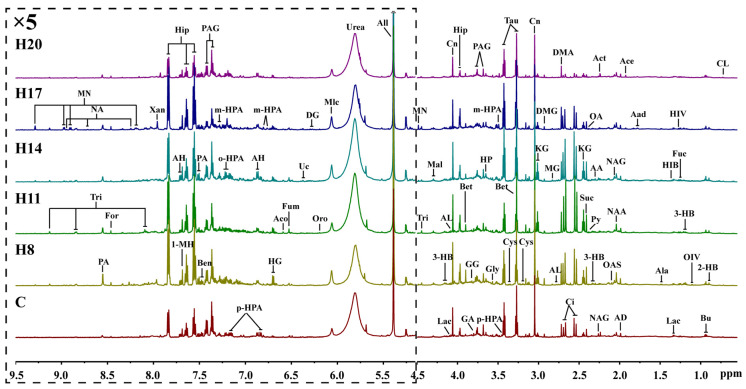
Representative 600 MHz ^1^H NMR spectra (δ0.5–5.1 and δ5.1–9.5) of intrauterine hypoxia rat urine samples obtained from control groups. CL: cholate; 2-HB: 2-Hydroxybutyrate; Bu: butyrate; OIV: 2-Oxoisovalerate; 3-HB: 3-Hydroxybutyrate; Fuc: fucose; HIV: 3-Hydroxyisovalerate; Lac: lactate; HIB: 2-Hydroxyisobutyrate; Ala: alanine; Aad: aminoadipate; Ace: acetate; AD: acetamide; NAA: N-Acetylaspartate; NAG: N-Acetylglutamate; OAS: O-Acetylglycoprotein; Act: acetone; AA: acetoacetate; Py: pyruvate; OA: oxaloacetate; Suc: succinate; KG: *α*-Ketoglutarate; Ci: citrate; DMA: dimethylamine; AL: 5-Aminolevulinate; MG: methylguanidine; DMG: N,N-Dimethylglycine; Cn: creatinine; Cys: cystine; Tau: taurine; Bet: betaine; p-HPA: para-Hydroxyphenylacetate; m-HPA: meta-Hydroxyphenylacetate; Gly: glycine; HP: hydroxypyruvate; PAG: phenylacetylglycine; GA: guanidoacetate; GG: glycylglycine; Hip: hippurate; Mal: malate; Tri: trigonelline; MN: 1-Methylnicotinamide; All: allantoin; Urea: urea; Mlc: maleicate; Oro: orotate; DG: deoxyguanosine; Uc: urocanate; Fum: fumarate; Aco: cis-aconitate; HG: homogentisate; AH: aminohippurate; o-HPA: ortho-hydroxyphenylacetate; Ben: benzoate; PA: picolinate; 1-MH: 1-Methylhistidine; Xan: xanthine; NA: nicotinamide; For: formate.

Metabolites produced in various organs are transported to the kidneys via blood, filtered through the kidneys, and finally excreted from the body via urine. Therefore, changes in urine metabolites can reflect the metabolic changes in the body. The NMR spectra of urine samples can also reflect the differences in metabolite concentrations in different groups (Figure 4). As the final product of the excretory system, the metabolites in urine samples are different from those in plasma, but most of them are the same. For example, different kinds of amino acids such as leucine, alanine, glycine, and valine were detected in serum NMR spectra, while some organic acids such as uric acid, phenylacetylglycine, and some metabolic waste products such as formic acid, hippuric acid, allantoin, and urea were detected in urine. Analysis of these typical metabolites can more specifically reflect the effect of hypoxia on maternal metabolites.

#### 2.2.2. Urine Metabolic Trajectories during Different Stages of Pregnancy under Hypoxic Conditions

**Figure 5:**
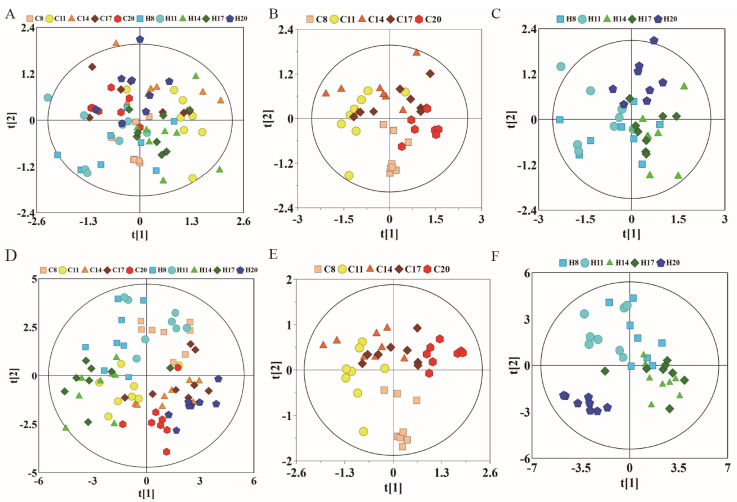
PCA and corresponding PLS-DA scores plots derived from ^1^H NMR spectra of urine samples obtained from different groups at different gestation period. (**A**) PCA score plots of control group and hypoxia group; (**B**) PCA score plots of control group; (**C**) PCA score plots of hypoxia group. (**D**–**F**) corresponding PLS-DA score plots of (**A**–**C**). (C: control group; H: hypoxia groups).

The NMR data of maternal urine samples at different stages of pregnancy were analyzed by PCA and PLS-DA to compare the metabolic status between hypoxia and control groups. As shown in Figure 5A, the first and second PCs accounted for 42.8% of the total variance. The data of D8 and D20 showed different degrees of overlap between the control and hypoxia groups, while there was a distinct separation trend on D11, D14, and D17, which is similar to the findings of plasma analysis. On PCA analysis, the first and second PCs accounted for 50.6% of the total variance in the control group (Figure 5B) and 44.8% of the total variance in the hypoxia group (Figure 5C). Compared to overall PCA, there was overlap of data in the two groups on D8, D14, and D17, with distinct separation on D8, D11, and D20. Moreover, there was a good distinction between D11 and D20. The most obvious change appeared on the 11th day of pregnancy, indicating that the most obvious metabolic changes occur on the 11th day. These results indicated similar metabolic changes between the hypoxia and control groups in the different stages of pregnancy. As shown in Figure 5D–F, total PLS-DA showed more distinct separation between the two groups, with partial overlap between hypoxia and control groups at any time-point of pregnancy. The results of PLS-DA showed significant differences between hypoxia and control groups during different stages of pregnancy; however, there was a partial overlap between D14 and D17, suggesting no obvious metabolic differences between these stages. To further analyze the metabolic changes induced by hypoxia, we subsequently compared the metabolite profile between the hypoxia and control groups at the same time-points of pregnancy.

#### 2.2.3. Urine Physiological and Metabolic Changes Induced by Hypoxia during Pregnancy

**Figure 6:**
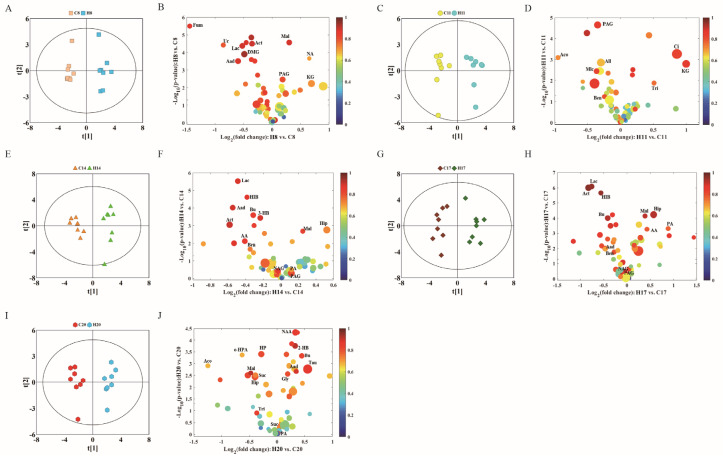
OPLS-DA scores plots (**left** panel) derived from ^1^H NMR spectra of urine samples and corresponding volcano plots (**right** panels) obtained from different groups and cross validation (**lower** panel) by permutation test (*n* = 200). (**A**,**B**) D8; (**C**,**D**) D11; (**E**,**F**) D14; (**G**,**H**) D17; and (**I**,**J**) D20. The color map shows the significance of metabolite variations between the two classes. Peaks in the positive direction indicate metabolites that are more abundant in the groups in the positive direction of first principal component. Consequently, metabolites that are more abundant in the groups in the negative direction of first primary component are presented as peaks in the negative direction. Keys of the assignments are shown in Figure 5.

Urine metabolite changes can be obtained from OPLS-DA analysis, which shows one-to-one correspondence with NMR. We conducted OPLS-DA of data pertaining to D8, D11, D14, D17, and D20 in the control and hypoxia groups (Figure 6A,C,E,G,I), combined with volcanic maps to identify significantly different metabolites (Figure 6B,D,F,H,J). The metabolite differences between the hypoxia and control groups are summarized in Table 2.

Through OPLS-DA and the correlation coefficient for each metabolite, we further summarized the metabolites which showed significant between-group differences. Compared with the control group, the hypoxia group showed significantly different levels of 29 urinary metabolites at different gestational time-points. The metabolite changes induced by hypoxia are summarized below: (1) The levels of citric acid and succinic acid (intermediates of TCA cycle) significantly decreased on D20, fumaric acid decreased on D8, while alpha-ketoglutarate significantly increased on D8 and D11. Malic acid significantly increased on D8 and D17, and decreased on D20. (2) Acetoacetic acid, 3-hydroxybutyric acid, and acetone levels showed a downward trend, which were similar to that in plasma, especially on D14 and D17, when these three ketones showed a significant decrease. (3) Glycine was significantly increased on D20. Phenylacetylglycine showed a significant decrease at all time-points, except on D20. Hippuric acid significantly increased on D14 and D17, while it significantly decreased on D20. Benzoic acid also significantly decreased in urine samples of D11, D14, and D17. (4) Lactic acid levels decreased on D8, D14, and D17. Pyridine formic acid decreased on D14, while it significantly increased on D17 and D20. (5) Uric acid significantly decreased on D8 and N-acetylglutamic acid significantly decreased on D17.

### 2.3. Metabolite Changes in Plasma and Urine and the Associated Pathways

**Figure 7:**
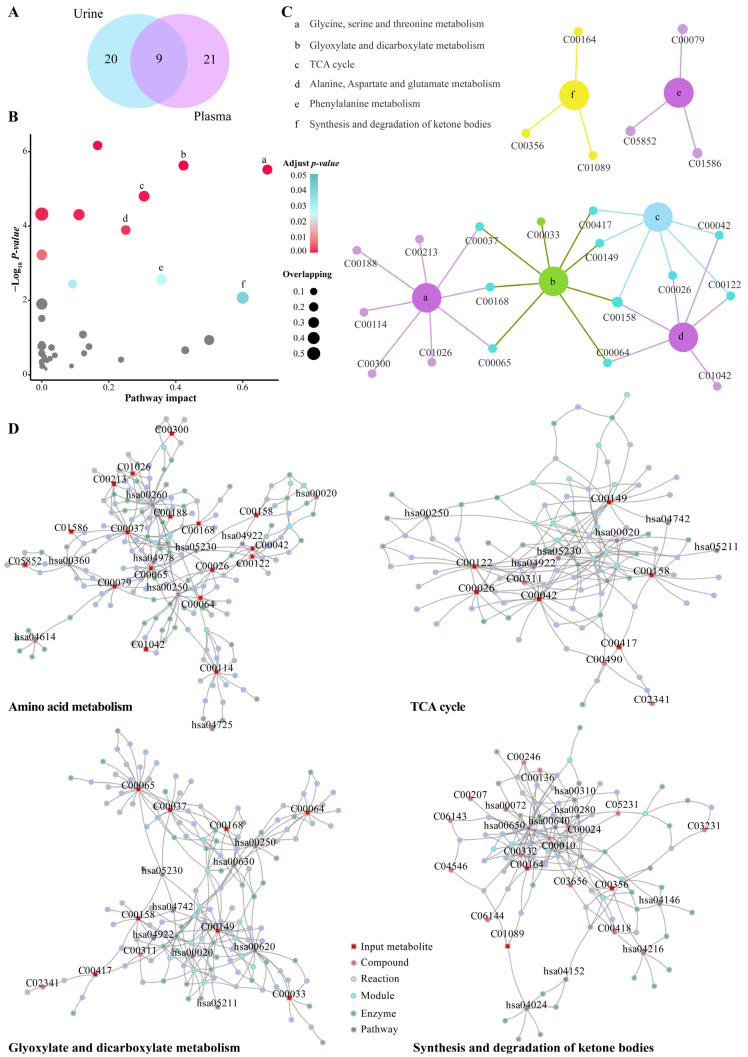
Pathway analysis and network diffusion analysis of differential metabolites. (**A**) Venn diagram of selected differential metabolites in urine and plasma. (**B**) Pathway analysis results of the MetaboAnalyst tools. The grey points indicate no significantly enriched pathway (adjusted *p* values > 0.05); the points of light blue to red indicate significantly enriched pathways (adjusted *p* values < 0.05); the size of the points indicates the number of overlapping metabolites in the pathway. (**C**) Plot showing the relevant differential metabolites contained in significantly enriched pathways. (**D**) Significantly enriched pathway-related metabolite–enzyme–reaction–module–pathway networks.

In the plasma and urine samples, we found 50 differential metabolites associated with the development of hypoxic rats during pregnancy (Figure 7A). Pathway analysis of these metabolites revealed six significantly enriched pathways, including the glycine, serine and threonine metabolism, alanine aspartate and glutamate metabolism, phenylalanine metabolism, the glyoxylate and dicarboxylate metabolism, synthesis and degradation of ketone bodies and TCA cycle (Figure 7B). The overlapping metabolites related to the enriched pathways are shown in Figure 7C, which was used to construct the metabolite–enzyme–reaction–module–pathway network. Figure 7D exhibits the multi-compound networks that are mainly involved in amino acid metabolism, TCA cycle, energy supply and ketone metabolism. This can contribute to a systematic understanding of the metabolic changes induced by hypoxia in pregnant rats and help inform other mechanism studies. We also observed significant changes in the lipid metabolites at different times of hypoxia in pregnant rats, which indicates that hypoxia can cause disorders of lipid metabolism.

**Figure 8:**
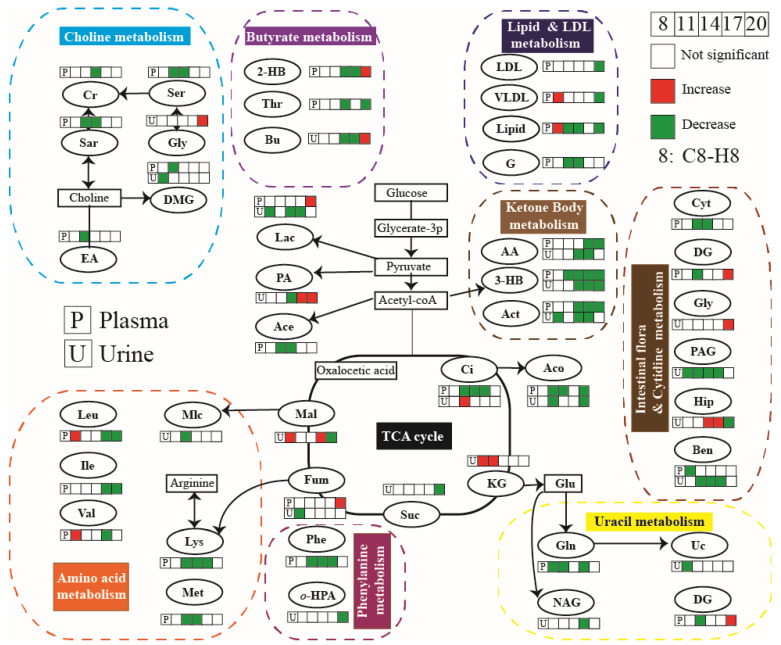
Metabolic pathway of maternal rat affected by hypoxia during pregnancy.

Furthermore, according to the typical differential metabolites and the significant enriched pathways, the integral metabolic pathways that may be affected by hypoxia during pregnancy (as shown in Figure 8) can be obtained by metabolic pathways of KEGG. The continuous chemical reaction and transformation of substrates and products in body fluid can reflect the abnormal anabolic and catabolic processes caused by hypoxia during pregnancy, including the metabolism disorders of glucose, lipid, amino acid, TCA cycle, urea cycle, and ketone.

## 3. Discussion

Metabolomics can characterize the changes in a broad spectrum of metabolites under different physiological or pathological conditions. Moreover, it can help identify biomarkers of early pregnancy complications, which can provide a basis for exploring the underlying pathogenetic mechanism. Indeed, this approach may provide a powerful tool for early detection of intrauterine hypoxia. In recent years, metabolomics has been widely used in the study of pregnancy-related diseases, such as gestational diabetes mellitus (GDM), preeclampsia, and pregnancy-induced hypertension with encouraging results [15,16,17,18]. In this study, we performed metabolomics studies in a rat model of gestational hypoxia. In particular, the metabolic profiles of plasma and urine were detected by 1H NMR and the metabolic trajectories of plasma and urine were further studied by multivariate statistical analysis. Our findings may help unravel the molecular mechanisms of the physiological effects of gestational hypoxia.

Hypoxia induces a multidimensional biological response involving complex intracellular networks and hypoxia-induced cellular signaling transduction [19]. Hypoxia during pregnancy has been shown to upregulate protein kinase C (PKC) and inhibit calcium-activated potassium channel (KCa)-mediated uterine artery relaxation [20]. Studies have also explored the cellular and molecular mechanisms of uterine vascular adaptation to chronic hypoxia during pregnancy. The results showed that chronic hypoxia during pregnancy induces abnormal expression patterns of steroid hormone receptor genes in uterine arteries, and increases myogenic response of uterine arteries through enhanced promoter methylation [21]. In this study, multivariate statistical analysis and metabolic pathway analysis of plasma and urine of pregnant rats showed significant hypoxia-induced metabolic changes, especially disorders of glucose metabolism, lipid metabolism, amino acid metabolism, and TCA cycle. Energy consumption in the setting of inadequate oxygen is known to stimulate anaerobic glucose degradation [22]. Lactic acid is the intermediate product of anaerobic glucose metabolism in the body, which can be used for energy compensation. Elevated plasma level of lactic acid indicates elevation of mitochondrial enzyme activity in plasma caused by hypoxia. Lipid metabolism entails both catabolism and storage of fat obtained from food or synthesized in liver. Lipids can be metabolized to supply energy in energy-deficient state. Studies have shown that abnormal lipid metabolism is closely related to many pathological conditions, such as fatty liver and hyperlipidemia [23]. Lipid metabolism disorder caused by hypoxia may cause liver function damage in pregnant rats. Elevated amino acid levels in plasma indicate that hypoxia may lead to the destruction of enzymes involved in amino acid metabolism, thus impeding amino acid reabsorption. The TCA cycle is the final metabolic pathway of the three major nutrients in the body, i.e., glucose, lipids, and amino acids. In this study, hypoxia induced significant changes in the levels of intermediate products of TCA, such as succinic acid, malic acid, fumaric acid, citric acid, and α-ketone glutaric acid. Hypoxia during pregnancy led to decrease in normal glycogen metabolism, alongside an increase in the metabolism of lipids and amino acids. Most of the metabolite levels showed a significant decrease, while most significant changes in plasma and urine metabolite levels were observed on D11. Hypoxia-induced metabolite changes during pregnancy may be related to the duration of hypoxia. We observed a cumulative effect of hypoxia on the metabolism with increasing duration of exposure; however, after a certain period of exposure, a compensatory effect was observed, which partially offset the effects of hypoxia on metabolism. 

This work employed an NMR-based metabolomics approach to study the metabolic variations in maternal plasma and urine using a rat model of gestational hypoxia. Metabolomics analysis showed that metabolites in plasma and urine samples were most significantly different between the control and hypoxia groups on the 11th day of pregnancy. The metabolic changes in maternal plasma and urine reflect both maternal physiological state and the fetal development and growth. Our results revealed changes in the levels of 30 metabolites in plasma and 29 metabolites in urine during different stages of pregnancy. Downregulation of acetoacetic acid, 3-hydroxybutyric acid, and acetone, both in plasma and urine, demonstrated that hypoxia suppressed ketone body metabolism during pregnancy. Moreover, the metabolites related to amino acid metabolism, TCA cycle, and energy supply were also prominently affected by hypoxia, mainly acetic acid, choline, citric acid, glutamine, isoleucine, lysine, and serine. These results suggested that gestational hypoxia primarily affects the amino acid metabolism, TCA cycle, energy supply, and ketone body metabolism, and this leads to the various physiological changes. Therefore, appropriate supplementation of ketone bodies and amino acids during pregnancy may be an effective means to prevent hypoxic injury during pregnancy.

In the present study, we identified distinct differences between the metabolites in the serum and urine samples of hypoxic pregnant rat models and control group at different gestational time-points. Pregnant rats exposed to hypoxia showed distinct differences in metabolites on days 8, 11, 14, 17, and 20 of exposure to hypoxia. Among them, the most notable metabolite changes were observed on D11. The difference between the model group and the control group decreased with the prolongation of hypoxia time, which indicates that the maternal pregnant rats may adapt to hypoxia after a certain period of exposure. Further, the shorter is the exposure to hypoxia during pregnancy, the more likely it is to derange the body fluid metabolism. This may be related to hypoxia-induced changes in placental metabolism [24,25]. For example, the oxygen tension during placental explant culture in vitro can dramatically alter the metabolic signatures. The human placenta is adapted for an initial hypoxic environment, then switches the metabolism to accommodate increased oxygenation in the second trimester due to extensive spiral artery remodeling. The intervillous oxygen tension increases from 2–3% at 8 weeks to 8.5% by 12 weeks of gestation with concurrent increased placental oxidative stress. Upregulation of placental antioxidant factors helps maintain redox homeostasis, and the metabolic profile of normal placental villus explants varies with oxygen culture conditions. Changes in hexadecanoic acid, erythritol, and 2-deoxyribose are particularly prominent. Our findings suggest that early screening and diagnosis of hypoxia can help inform interventions to improve the poor perinatal prognosis.

This study suggests that metabolomics study of the effect of gestational hypoxia can provide insights into the occurrence, development, and prognosis of obstetric disorders, including both maternal and fetal disorders. Considering the current prenatal screening methods, it is clear that metabolomics can generate new insights into the biological and physio/pathological processes. Moreover, it can help identify biomarkers of early pregnancy complications, which can provide a foundation for studying the underlying pathogenetic mechanisms and further metabolomics research in the field of obstetrics. In this perspective, this approach may provide a powerful tool for prenatal screening of intrauterine hypoxia and a foundation for a better understanding of pathological mechanisms.

## 4. Materials and Methods

### 4.1. Ethics Statement

The study was performed in accordance with the relevant guidelines and regulations of the Xiamen University. All animal studies were approved by the Animal Ethics Committee of Quanzhou Medical College Laboratory Animal Center (laboratory animal license number: SYXK (Fujian) 2009-0004, certificate: 2012001).

### 4.2. Animal Model Establishment

#### 4.2.1. Pregnant Rat Model

Eighty female Sprague–Dawley (SD) rats (weight 200–250 g) and 80 male SD rats (weight: 300–350 g) with no history of mating were purchased from the Shanghai Laboratory Animal Center, CAS (SLACCAS) (License No. SCXK (Shanghai) 2012-0002, Certificate No. 2007000561158). All rats were housed in a controlled environment in SPF laboratory animal room (temperature: 18–25 °C, humidity 40–75%, 12-h light-dark cycle) and provided ad libitum access to water and food. After 3–5 days of adaptive feeding in the laboratory animal center, male and female rats (ratio of 1:1) were housed in the same cage at 16:00 h to allow for mating. A tray with a clean and waterproof surgical towel was placed under the cage. At 8:00 h on the next day, female rats were examined for the presence or absence of vaginal plug. The vaginal plug consists of milky-white (sometimes partially or completely pink), rat-shaped, solid colloidal particles. Presence of vaginal plug was considered indicative of successful pregnancy (day 0). Rats without vaginal plug were allowed to continue to mate until the appearance of vaginal plug. Pregnant rats were removed and housed in separate cages. Finally, 80 healthy SD pregnant rats were randomly divided into normoxic control group and hypoxia group (*n* = 40 in each group).

#### 4.2.2. Establishment of Chronic Hypoxia Animal Model

Hypoxia group (Group H): Hypoxia group was established as described previously [12]. In brief, from day 7 to day 20 of pregnancy, rats (*n* = 40) were placed in a Plexiglas chamber (volume, 140 L) twice a day for anoxia, 4 h per time, until childbirth (Day 20) with free access to food and water. A portable oxygen analyzer (S-450; IST-AIM) was used to monitor the oxygen concentration of the chamber (calibrated daily). Oxygen concentration was maintained at 12 ± 0.1% by continuous infusion of a mixture of nitrogen gas and compressed air. The expired CO_2_ was eliminated by circulating chamber air through soda lime and the water contained in the expired gas was trapped in a chilled glass tank. The pores in the anoxic box helped maintain a balance between the air pressure and atmospheric pressure in the box. The carbon dioxide concentration in the box was always <3%, and the temperature was maintained at room temperature. After one-hour hypoxia every day, 8 pregnant rats in each group were randomly selected for arterial blood gas analysis to verify successful establishment of the model.

Control group (Group C): Forty Sprague–Dawley pregnant rats in the control group were placed in the hypoxic chamber but were not exposed to hypoxia as the chamber was continuously ventilated. The other conditions were identical to those in the hypoxia group.

### 4.3. Sample Collection

On the 8th day (D8), 11th day (D11), 14th day (D14), 17th day (D17), and 20th day (D20) of gestation, urine and blood samples were obtained from eight randomly selected pregnant rats in group C and group H. The data were recorded as C8, C11, C14, C 17, and C20, respectively, in the control group and as H8, H11, H14, H17, and H20, respectively, in the hypoxia group.

Blood samples were drawn into sterile tubes with heparin, kept at 4 °C for 5 min, and then centrifuged at 10,000 rpm for 10 min to separate plasma from blood cells. The plasma was pipetted into 1 mL sterile microtubes. Urine samples were collected in metabolic cage and transferred to 5 mL microtubes with NaN_3_ at 4 °C. All samples were stored at −80 °C until analysis.

### 4.4. Nuclear Magnetic Resonance (NMR) Analysis

^1^H NMR measurements were performed on all plasma and urine samples using a 600 MHz NMR spectrometer (Bruker Corporation, Karlsruhe, Germany) equipped with a cryogenic probe. The proton resonance frequency was 600.13 MHz and the detection temperature was 298 K.

#### 4.4.1. Plasma Samples

Frozen plasma was thawed on ice and 200 μL plasma samples were mixed with 400 µL phosphate buffer solution (90 mM/L, pH 7.4), and then centrifuged at 4 °C, 6000 rpm for 10 min. A quantity of 550 μL supernatant was used for NMR analysis. Spectra of plasma samples were obtained using the NOESYPR-CPMG pulse sequence (echo time: 70 ms; relaxation delay (RD): 2.0 s). NOESYPR sequence (delay-90°-t_1_-90°-t_m_-90°-acquisition) can suppress water peak; delay time t_1_ was 2 μs and delay time t_m_ was 120 ms. The signal accumulation was 32 times, the sampling points were 32K, the spectrum width was 10K, the sampling time was 2.73 s, and the relaxation delay (RD) was 4 s.

#### 4.4.2. Urine Samples

Urine samples were thawed on ice at 4 °C and 200 μL samples were mixed with 400 μL buffer solution (K_2_HPO_4_/Na_2_HPO_4_, 1.5 M, pH 7.4, containing 0.05%TSP, 99.9%D_2_O) and then centrifuged at 4 °C, 10,000 rpm for 10 min. 550 μL supernatant was used for NMR analysis. All samples were placed at 4 °C before NMR analysis. Spectra of urine samples were obtained using the NOESYPR1D sequence (RD-90°-τ_1_-90°-τ_m_-90°-ACQ) (spectrum width: 10K; t_1_: 5 μs; t_m_: 80 ms; RD: 2 s; sampling points: 16K; signal accumulation: 64 times).

### 4.5. Spectra Processing and Statistical Analysis 

#### 4.5.1. Spectra Processing

The free induction decay (FID) signals of plasma and urine samples were imported into MestReNova (V9.0, Mestrelab Research S.L.) software. The ^1^HNMR spectra were Fourier-transformed, baseline-corrected, phase-adjusted, aligned, and calibrated. All spectra in Fourier transform were multiplied by exponential window function of broadening factor 1 Hz (plasma) and 0.3 (urine sample) to improve the signal-to-noise ratio.

The plasma NMR spectrogram was calibrated with the double peak of lactic acid at 1.33 ppm. The urea peak 6.0–5.5 ppm, the residual water peak 5.23–4.54 ppm, and its adjacent peaks were removed. The remaining peak 9.0–0.5 ppm was integrated with an integral spacing 0.002 ppm. Urine NMR spectrogram was calibrated with a single peak of the intensity of the internal standard (TSP) at 0.00 ppm. The urea peak 6.00–5.55 ppm and the residual water peak 5.10–4.50 ppm, and its adjacent peaks were removed. The remaining peak 9.5–0.5 ppm was integrated with an integral spacing 0.005 ppm.

#### 4.5.2. Multivariate Analysis

The integrated data were imported into SIMCA-P software (version 14.0, Umetrics AB, Umeå, Sweden) for principal component analysis (PCA), partial least squares-discriminant analysis (PLS-DA), and orthogonal partial least squares-discriminant analysis (OPLS-DA). PCA was used to summarize the data distribution and identify the potential outliers using mean center scaling. PLS-DA was used to assess the correlation between NMR data (X variable) and other variables (Y variables) in various groups. The scale of PLS-DA and OPLS-DA data was converted to Par (Pareto scaling). OPLS-DA was used to maximize the differences between different groups within the model, and to perform cross validation of the model, permutation test (*n* = 200), and cross-validation analysis of variance (CV-ANOVA).

Metabolites with significant between-group differences were screened out after pattern recognition using OPLS-DA, and then color volcanic maps based on OPLS-DA were drawn to show the differences of metabolites. Four-dimensional information contained in the volcanic map, i.e., r (correlation coefficient), VIP (variable projection importance), fold-change (multiple variation of relative concentration between groups) and *p* value of *t*-test, improved the reliability of the screened differential metabolites. Each scatter represents a metabolite in body fluids; the *x*-axis and *y*-axis represent *log*2 (fold change) and *−log*10 (p), respectively. Positive points on the *x*-axis indicate that the metabolite concentrations in the hypoxic group were higher than those in control group on paired comparison. The color of the dots was determined by the absolute value of correlation coefficient |r|. Warm color represents strong correlation between groups, cold color indicates weak correlation between groups. The size of the dots was determined by VIP values; larger dots correspond to metabolites with higher VIP values, while smaller dots correspond to metabolites with lower VIP values. Therefore, the four-dimensional volcano map provided comprehensive information about the differences in metabolomics among different groups. All volcanic maps were generated by the script written by MATLAB (downloaded from http://www.mathworks.com, (accessed on 9 June 2021)).

### 4.6. Pathway Analysis

Pathway analysis was performed using MetaboAnalyst (http://www.metaboanalyst.ca/) to further determine the deranged metabolic pathways during the development of chronic hypoxia during pregnancy. These pathways and differential metabolites were then mapped to upstream compounds such as enzymes, other metabolites and reactions by means of network diffusion analysis [13]. This process was done using the FELLA and igraph packages of R software [14]. 

## Figures and Tables

**Table 1:** Differences in plasma metabolism between hypoxia group and control group.

Metabolites	C8–H8		C11–H11		C14–H14		C17–H17		C20–H20	
	r	Fold Change	r	Fold Change	r	Fold Change	r	Fold Change	r	Fold Change
3-Hydroxybutyrate	/	0.998	−0.821	0.715	−0.865	0.548	−0.881	0.415	−0.797	0.755
Acetoacetate	/	1.23	/	0.791	/	0.522	−0.797	0.475	−0.712	0.704
Acetate	/	1.021	−0.688	0.845	−0.792	0.856	/	0.844	/	0.925
Acetone	/	1.416	/	0.972	−0.822	0.743	−0.814	0.446	−0.706	0.829
Benzoate	−0.675	0.907	/	0.905	/	0.92	/	0.58	/	1.08
cis-Aconitate	0.738	0.974	/	1.09	/	1.237	/	1.578	/	0.208
Choline	/	0.91	−0.886	0.923	−0.72	0.82	/	0.927	−0.777	1.011
Citrate	/	0.862	−0.785	0.848	−0.804	0.818	−0.791	0.954	/	0.973
Creatine	/	0.818	/	0.843	−0.689	0.734	/	0.857	/	1.148
Cytidine	/	0.899	−0.698	0.863	−0.722	0.728	/	1.349	/	1.082
Deoxyguanosine	/	0.975	−0.676	0.776	/	0.724	/	1.543	0.804	1.786
N,N-Dimethylglycin	/	0.987	−0.835	0.846	/	0.871	/	0.972	/	1.006
Ethanolamine	/	0.917	−0.782	0.918	/	0.996	/	1.185	/	1.122
Fumarate	/	0.551	/	0.129	/	0.126	/	0.043	0.741	1.729
Glutamine	−0.718	0.961	−0.815	0.919	/	0.925	−0.831	0.894	/	1.053
Glycerol	/	0.926	−0.781	0.918	−0.853	0.862	/	0.932	/	1.038
Isobutyrate	/	1.054	/	0.862	−0.782	0.807	−0.693	0.8	/	1.055
Isoleucine	/	1.08	/	0.966	/	0.968	−0.831	0.872	−0.827	0.906
Lipid	0.726	/	−0.764	/	−0.827	/	/	/	−0.702	/
VLDL	0.681	/	/	/	/	/	/	/	−0.909	/
LDL	/	/	/	/	/	/	/	/	−0.884	/
Lactate	/	0.927	/	0.793	/	1.036	/	0.811	0.834	1.132
Leucine	0.691	1.087	/	0.996	/	0.995	−0.805	0.815	−0.8	0.979
Lysine	/	1.096	−0.92	0.818	−0.774	0.887	−0.809	0.784	/	0.934
Methionine	/	0.944	−0.916	0.839	−0.797	0.909	/	0.902	/	1.002
Phenylalanine	/	0.969	−0.785	0.892	−0.778	0.85	−0.766	0.745	/	0.967
Sarcosine	/	0.971	−0.718	0.898	−0.772	0.916	/	0.956	/	1.007
Serine	/	0.95	−0.686	0.896	−0.764	0.855	/	0.972	/	0.947
Threonine	/	1.01	/	0.964	−0.907	0.834	/	0.982	−0.754	0.961
Valine	0.844	1.108	/	0.915	/	1.014	−0.817	0.874	/	0.994
Cells are color-coded according to the fold-change; red indicates increased and blue indicates decreased in each group. Color bar:										
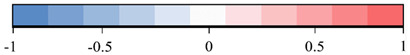										

OPLS-DA coefficients derived from the NMR data of metabolites in serum samples obtained from different groups. Correlation coefficients, positive and negative signs indicate positive and negative correlation in the concentrations, respectively. The correlation coefficient of |r| > 0.666 was used as the threshold for statistical significance based on the discrimination significance at the level of *p* = 0.05 and *df* (degree of freedom) = 7. “/” means the correlation coefficient |r| is less than 0.666.

**Table 2:** Differences in urine metabolism between hypoxia group and control group.

Metabolites	C8–H8		C11–H11		C14–H14		C17–H17		C20–H20	
	r	Fold Change	r	Fold Change	r	Fold Change	r	Fold Change	r	Fold Change
1-Methylhistidine	−0.807	0.867	/	0.862	/	1.058	/	1.044	/	0.792
2-Hydroxybutyrate	/	0.949	/	1.076	−0.841	0.976	−0.89	0.939	0.92	1.255
3-Hydroxybutyrate	/	0.978	/	0.981	−0.892	0.851	−0.89	0.783	−0.853	1.045
Acetoacetate	/	0.922	/	1.004	−0.861	0.753	−0.867	0.67	/	1.165
Acetone	−0.901	0.783	/	1.03	−0.903	0.668	−0.965	0.563	/	1.213
Allantoin	/	1.071	−0.673	0.814	/	1.047	/	1.195	/	0.911
Aminoadipate	−0.887	0.661	/	1.106	−0.898	0.685	−0.814	0.724	/	1.259
Benzoate	/	0.84	−0.805	0.846	−0.746	0.786	−0.806	0.825	/	1.027
Butyrate	/	0.777	/	0.947	−0.849	0.805	−0.922	0.751	0.829	1.362
cis-Aconitate	/	1.152	−0.724	0.518	/	1.284	/	0.65	−0.678	0.422
Citrate	/	1.86	0.831	1.815	/	1.292	/	0.882	/	0.817
Fumarate	−0.882	0.367	/	0.672	/	0.543	/	0.45	/	1.13
Glycine	/	1.12	/	1.082	/	1.208	/	1.064	0.811	1.143
Hippurate	/	1.035	/	1.053	0.729	1.437	0.923	1.487	−0.807	0.76
Hydroxypyruvate	/	1.088	/	0.909	/	1.235	/	1.123	−0.812	0.822
Lactate	−0.897	0.695	/	1.201	−0.888	0.713	−0.956	0.589	/	0.986
Malate	0.891	1.23	/	0.905	/	1.189	0.917	1.308	−0.915	0.72
Maleicate	/	0.937	−0.835	0.778	/	0.969	/	1.221	/	0.972
N,N-Dimethylglycine	−0.942	0.713	/	1.14	/	0.692	/	0.847	/	1.932
N-Acetylaspartate	0.809	1	/	0.965	/	1.095	/	1.114	0.87	1.08
N-Acetylglutamate	/	0.916	/	1.141	/	0.979	−0.926	0.965	/	1.125
ortho-Hydroxyphenylacetate	/	0.902	/	0.953	/	0.909	/	0.905	−0.686	0.647
Phenylacetylglycine	0.786	1.178	−0.845	0.787	−0.813	1.086	−0.858	0.971	/	0.905
Picolinate	/	0.934	/	1.014	−0.706	1.087	0.789	1.346	0.785	1.011
Succinate	/	1.151	/	1.35	/	1.16	/	0.908	−0.736	0.764
Taurine	/	0.827	/	0.761	/	0.884	/	0.984	0.868	1.467
Trigonelline	/	1.176	0.764	1.425	/	1.428	/	1.875	−0.842	0.777
Urocanate	−0.793	0.552	/	1.012	/	0.796	/	0.907	/	0.913
α-Ketoglutarate	0.718	1.612	0.859	1.998	/	1.29	/	0.879	/	0.981
Cells have been color-coded according to the fold-change; red indicates increased and blue indicates decreased in each group. Color bar:										
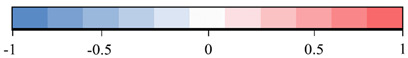										

OPLS-DA coefficients derived from the NMR data of metabolites in urine. correlation coefficients, positive and negative signs indicate positive and negative correlation in the concentrations, respectively. The correlation coefficient of |r| > 0.666 was used as the cutoff value for statistical significance based on the discrimination significance at the level of *p* = 0.05 and *df* (degree of freedom) = 7. “/” means the correlation coefficient |r| is less than 0.666. multiplicity: s, singlet; d, doublet; t, triplet; q, quartet; dd, doublet of doublets; m, multiple.

## Data Availability

The datasets generated and analyzed in the present study are available from the corresponding author upon reasonable request.
